# Improving neonatal health with family-centered, early postnatal care: A quasi-experimental study in India

**DOI:** 10.1371/journal.pgph.0001240

**Published:** 2023-05-25

**Authors:** Seema Murthy, Shirley Du Yan, Shahed Alam, Amit Kumar, Arjun Rangarajan, Meenal Sawant, Huma Sulaiman, Bhanu Pratap Yadav, Tanmay Singh Pathani, Anand Kumar H. G., Sareen Kak, Vinayaka A. M., Baljit Kaur, Rajkumar N., Archana Mishra, Edith Elliott, Megan Marx Delaney, Katherine E. A. Semrau

**Affiliations:** 1 Aurora Health Innovations, Bengaluru, India; 2 Noora Health, San Francisco, California, United States of America; 3 Data Science, ShriSankhyam Analytics and Research LLP, New Delhi, India; 4 Department of Health and Family Welfare, Government of Punjab, Chandigarh, India; 5 Department of Health and Family Welfare, Government of Karnataka, Bengaluru, India; 6 Directorate of Public Health & Family Welfare, National Health Mission, Bhopal, Madhya Pradesh, India; 7 Ariadne Labs, Harvard T.H. Chan School of Public Health/Brigham and Women’s Hospital, Boston, Massachusetts, United States of America; 8 Department of Medicine, Harvard Medical School, Boston, Massachusetts, United States of America; University of Regina, CANADA

## Abstract

Despite the global decline, neonatal mortality rates (NMR) remain high in India. Family members are often responsible for the postpartum care of neonates and mothers. Yet, low health literacy and varied beliefs can lead to poor health outcomes. Postpartum education for family caregivers, may improve the adoption of evidence-based neonatal care and health outcomes. The Care Companion Program (CCP) is a hospital-based, pre-discharge health training session where nurses teach key healthy behaviors to mothers and family members, including skills and an opportunity to practice them in the hospital. We conducted a quasi-experimental study to assess the effect of the CCP sessions on mortality outcomes among families seeking care in 28 public tertiary facilities across 4 Indian states. Neonatal mortality outcomes were reported post-discharge, collected via phone surveys at four weeks postpartum, between October 2018 to February 2020. Risk ratios (RR), adjusting for hospital-level clustering, were calculated by comparing mortality rates before and after CCP implementation. A total of 46,428 families participated in the pre-intervention group and 87,305 in the post-intervention group; 76% of families completed the phone survey. Among the 33,599 newborns born before the CCP implementation, there were 1386 deaths (NMR: 41.3 deaths per 1000 live births). After the intervention began, there were 2021 deaths out of 60,078 newborns born (crude NMR: 33.6 deaths per 1000 live births, RR = 0.82, 95% CI: 0.76, 0.87; cluster-adjusted RR = 0.82, 95% CI: 0.71, 0.94). There may be a substantial benefit to family-centered education in the early postnatal period to reduce neonatal mortality.

## Introduction

Neonatal mortality disproportionately affects families in low-to-middle income countries (LMICs) [1, 2]. With 21.7 deaths per 1000 live births, India has the third-highest Neonatal Mortality rate (NMR) globally [3]. India’s NMR has declined sharply from 1990 (57.4 deaths) [4]. Current strategies to improve neonatal survival include increasing institutional deliveries, increasing coverage of care, creating structured infrastructure across communities for the care of at-risk babies, and improving healthcare access have reached a ceiling effect [5]. To further improve neonatal survival, there is a need for different approaches, and more innovative targeted interventions [6, 7].

Most deaths can be prevented by providing Continuum of Care (CoC) in a structured way from pregnancy to the post-delivery period [8]. Interventions that take a lifecycle approach addressing the needs of the woman before, during, and after her pregnancy, as well as infant and childcare, are required. Although institutional deliveries have nearly doubled in India over the past decade [9], insufficient follow-up visits create gaps in postnatal care. According to the National Family Health Survey (NFHS-4), only 39% of pregnant women in India had completed CoC between 2015 and 2016 [8], including the utilization of 4 + antenatal care, institutional delivery, postnatal care, and immunization. In LMICs, only 58% of babies complete the postnatal visit within two days after birth [10]. Besides insufficient follow-up care, caregivers often lack knowledge of correct practices for providing care and/ or identifying critical situations where additional and immediate care is required for the neonates [10]. It is possible that lack of required follow-up visits and absence of knowledge for adequate postnatal care can have a combined effect on the baby and/or mother’s health increasing the risk of mortality and morbidity.

Although certain healthcare interventions can only be provided by skilled healthcare personnel, there are many practices that caregivers can and should do on an ongoing basis, but the caregivers need to be first equipped through training to do so [10]. Postnatal training is an important strategy to increase the skills of caregivers and families. With the increase in institutional deliveries, hospital stays provide a window of opportunity to equip caregivers with the necessary awareness and skills. A hospital-based program that can systematically train caregivers could potentially address this gap in caregiver knowledge, leading to improved care practices and health outcomes.

### Program description

**Fig 1:**
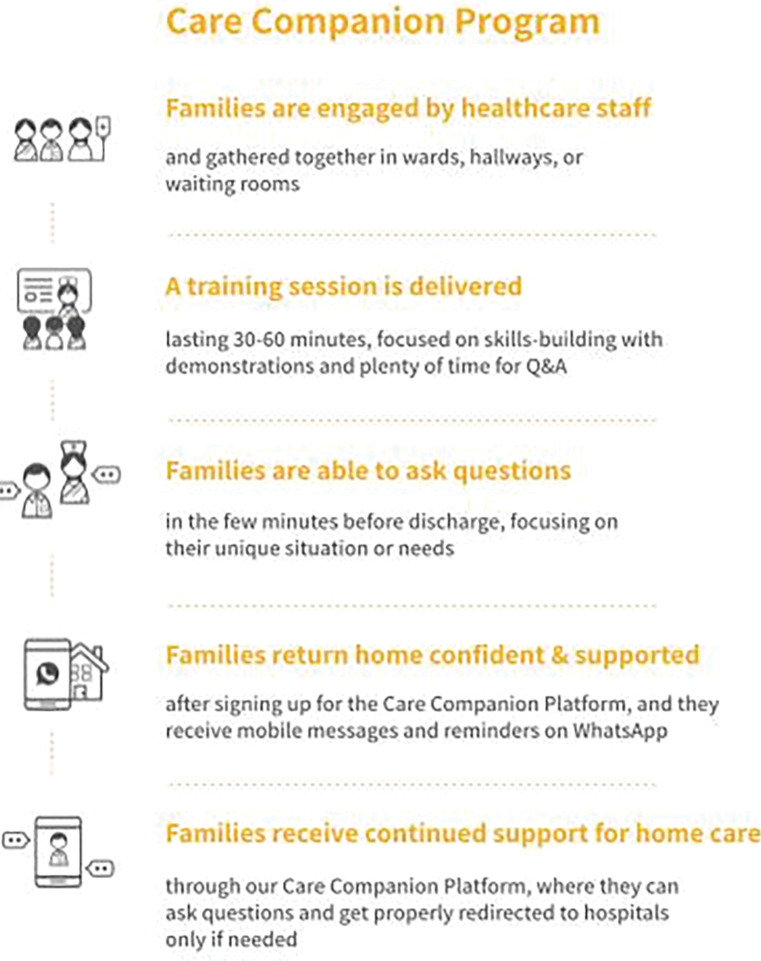
Description of the hospital experience without and with CCP.

The Care Companion Program (CCP) trains families on essential maternal and neonatal care during their hospital stay. Noora Health, a non-profit organization, supports the design of CCP, which is being implemented with local partners across district hospitals in seven states of India [11]. The CCP uses an evidence-based curriculum for postnatal counseling created using a human-centered design process to promote behavior change. It includes behaviors for improving neonatal and maternal health [12], such as early and exclusive breastfeeding; hygienic cord care; hand hygiene; skin-to-skin care (kangaroo mother care); burping; healthy diet for the mother; the importance of follow-up and immunizations; and recognition of all danger signals, particularly respiratory infections, diarrhea, and jaundice. Information is taught using visual aids and materials tailored to the cultural practices of local communities and patient populations [12]https://www.zotero.org/google-docs/?SXBXbC. Through two to three days of training of trainers, nurses and health educators receive training on health behavior change and skills to effectively communicate health information by engaging their audiences. They are provided teaching aids that include videos played on television monitors in the postnatal wards, flipcharts, demonstration tools (e.g. dolls) used for role-playing, and hand-outs. Using these varied materials, nurses and health educators conduct group classes for families in postnatal wards for approximately 30 minutes per session. CCP sessions are conducted 3–5 times per week at each postpartum ward, depending on a hospital’s preference. Additionally, families receive post-discharge follow-up through a dedicated WhatsApp-based platform, which reiterates key behavior change messages. Further, this approach allows for two-way communication, providing families the opportunity to ask questions and receive guidance from Noora Health’s affiliate support staff (both doctors and nurses). Initial findings from two hospitals showed promising results for increased family behavior change and reduction in preventable complications and facility readmissions [13]. Through the CCP, caregivers are educated on right caregiving practices and equipped with skills to provide quality care. We expect that this would help families adopt better care practices which can thereby impact the survival and wellbeing of the neonate. Please see Fig 1 for more details.

A large evaluation study assessing the impact of the CCP intervention on knowledge, practice, and outcomes among families seeking childbirth care in 28 facilities across 4 Indian states has been underway since 2019. The data collection for the main study was temporarily paused due to the COVID-19 pandemic from March, 2020 to June, 2022. Here, we are reporting the findings from the mid-point analysis assessing the early impact of the CCP on neonatal mortality, prior to the COVID-19 pandemic.

## Methods

### Procedures

A quasi-experimental study design was used to assess the impact of CCP on maternal and neonatal health outcomes. Data collected from October 2018 to February 2020 were analyzed for mortality outcomes in this interim analysis. Among the 28 study hospitals, 24 district-level hospitals (DHs) were selected randomly from 91 high and low delivery-load hospitals in three Indian states, Karnataka (KA), Punjab (PB), and Madhya Pradesh (MP), in which CCP would be implemented. In Maharashtra (MH), four government medical college hospitals were also included.

Trained investigators collected contact details of the mothers in the hospital, after delivering the baby, and just before discharge. Among eligible participants, consent for the study was asked for in the hospital, and reconfirmed at the time of the survey call. In the event of a maternal or neonatal death prior to discharge; maternal age less than 18 years; or no consent provided; mothers/families were excluded from the study. At the end of the neonatal period (i.e. 4 weeks post discharge), the study staff called the consenting and eligible mothers to learn about vital status and neonatal care behaviors. Those who were not reached or those who refused the interview were marked as incomplete. At the beginning of the follow-up phone call, vital status of the mother and newborn was collected. Maternal deaths during pregnancy, during hospital delivery before discharge, abortions, home deliveries, or those who died after 28 days and before 42 days are not included. In cases of a maternal or neonatal death, information around neonatal care behaviors or sociodemographic data were not collected due to ethical concerns of continuing the interview. As a result, it is not possible to adjust our statistical analysis by sociodemographic characteristics.

Data from the pre-intervention period prior to the CCP launch date at each facility were compared to post-intervention data, which was collected in a continuous fashion after the CCP launch date at that facility. We assessed the effect of the program with an intention-to-treat approach, irrespective of whether individuals in the intervention facilities were trained or not. No time elapsed between when the program started and data collection began. We compared the post-discharge mortality for mothers and neonates in the pre-intervention period to the post-intervention period.

### Sample size

In the original evaluation design, we focused on behavior change around neonatal care. Assuming an 80% power and an alpha level of 0.05, it was hypothesized that the rate of unsafe cord care would drop by 21%. Assuming a classic pre-post single setting sample calculation, it was concluded that 330 per facility would suffice to detect statistically significant differences (or 9,240 total). In a post hoc sample size calculation, we estimated the power of this analysis to detect differences in neonatal mortality rates (NMR) and maternal mortality rates (MMR) using R version 4.1. With the current sample size, the study has 98% power to detect the estimated difference in NMR of 8.7 deaths per 1000 live births between the pre- and post-CCP session groups. Additionally, the current sample size has 70% power to detect the estimated difference in MMR of 6.9 deaths per 100,000 delivered mothers.

### Analysis

We used logistic regression models to assess the impact of the intervention (pre versus post) on neonatal and maternal mortality. Our dataset was limited to those who reported outcomes during a follow-up call. The regression models reporting risk ratios and risk differences were adjusted for hospital-level clustering; however, we were unable to adjust for sociodemographic data as this information was not collected from families who experienced a death. All analyses were completed with STATA 16 [14].

Additionally, we compared the rate of change between pre and post CCP against the general NMR trend within India. General NMR trends for India from 2012–2019 is sourced from the World Bank [15].

### Ethical review

We received IRB approval for primary data collection from ACE Ethics Committee and SPECT based in Bangalore, India and Delhi, India respectively. The primary data with identifiable information were collected and maintained in India; deidentified data were shared with collaborators for analysis. For this secondary analysis (DCGI Reg. No. ECR/141/Indt/KA/2013), Harvard University Institutional Review Board (Protocol # IRB19-1140) deemed the study exempt. All participants underwent an oral consent process at the hospital and again during the follow-up phone call, which was audio recorded.

## Results

### Enrollment & follow-up

**Fig 2:**
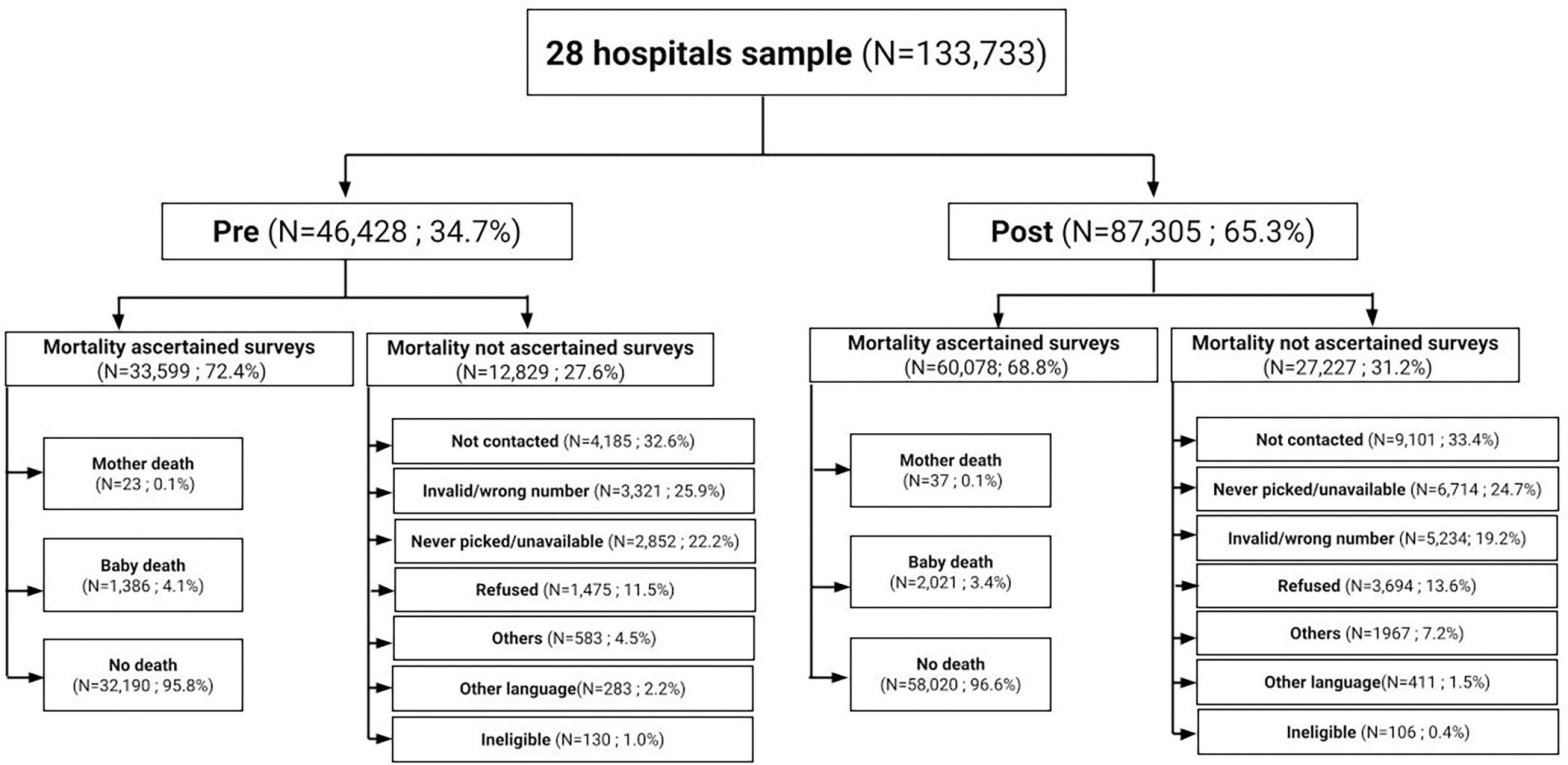
Flowchart and sample size.

We collected phone numbers from a total of 133,733 families in the hospital, with 46,428 from the pre-intervention period and 87,305 from the post-intervention period (Fig 2). We successfully contacted and ascertained mortality outcomes in 33,599 (72.4% response rate) of the pre-intervention group and 60,078 (68.8% response rate) of the post-intervention group. The overall response rate was 70.6%. Among the non-responders, the primary reasons for non-response in both study periods were due to lack of phone line connection and invalid phone number. Under ineligibility, the most frequent criterion was that the baby was delivered to a different facility and then shifted to the current hospital.

#### Characteristics of families without mortality

Among families that did not experience neonatal or maternal death, socio-demographic details are shown in S1 Table to demonstrate the overall population attending care in these facilities. The pre- and post-CCP groups differed on mother’s age, education level, type of delivery, number of babies delivered, and length of stay. Mothers in pre-CCP group were younger (ages < = 25 years), more educated, and mostly had singleton deliveries. A statistically significant difference was also observed in length of stay among two groups. Typically, mothers in pre-CCP group spent less time (less than 48 hours) in the hospital compared to those in the post CCP group (48 hours to 7 days).

#### Mortality

Among the 33,599 newborns before the Program, there were 1386 deaths (NMR: 41.3 deaths per 1000 live births). After the intervention began, there were 2021 deaths out of 60,078 newborns born (NMR: 33.6 deaths per 1000 live births). The unadjusted Risk Ratio (RR) for neonatal mortality in the pre-intervention compared to post-intervention group was 0.82 (95% CI:0.76, 0.87). After adjusting hospital clustering, the risk ratio was similar RR = 0.82, 95% CI: 0.71, 0.93 (S2 Table).

Reduction in maternal mortality was not significant. In the pre-intervention period, 23 maternal deaths of 33,599 births occurred after facility discharge and post-intervention 37 maternal deaths out of 60,078 births occurred (S3 Table). The unadjusted and hospital-level clustering adjusted risk ratio for maternal mortality per 100,000 live births, in the Post versus Pre group was 0.90 (95% CI: 0.53, 1.51) and 0.87 (95% CI:0.53, 1.43) respectively.

In S4 Table, neonatal mortality adjusted for state and hospital-level clustering, and crude model by the state are reported. None of the state-specific analyses had statistically significant findings. In the overall model adjusted for state and hospital level clustering, the adjusted risk ratio was 0.93 (95% CI 0.82, 1.06). For Punjab, the risk ratio showed a slight increase in neonatal mortality (1.06, 95% CI: 0.84, 1.33). For the rest of the states, the risk ratio for neonatal deaths showed a decreased trend: Karnataka RR = 0.88, 95% CI: 0.69, 1.12; Maharashtra RR = 0.73, 95% CI 0.49, 1.08; Madhya Pradesh RR = 0.97, 95% CI 0.83, 1.13.

**Fig 3:**
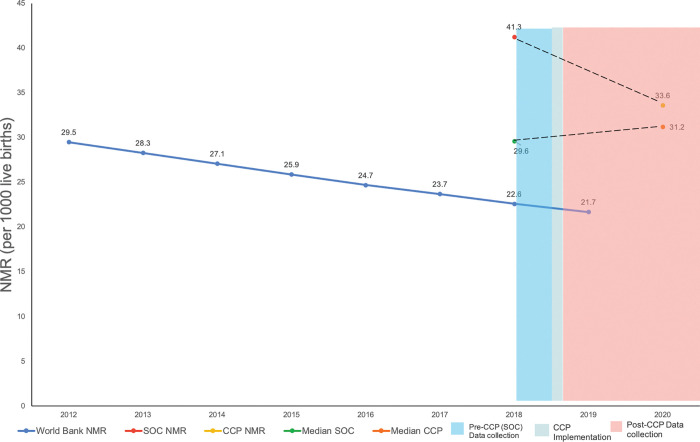
Associated impact of CCP on neonatal mortality.

Fig 3 shows the NMR trendline between pre- and post-implementation of CCP compared to the larger trendline for India, as reported by the World Bank [15]. This trend is consistent with nationally collected data over a similar timeframe (S5 Table). Historically, NMR reduced by 9 per 1000 births (39 to 30 per 1000 births), over a much longer 10-year time frame, between NFHS-3 and 4 (2005–06 and 2015–16 respectively) [15]. In the 1.5 years of program implementation, this translates to 9.2 per 1000 births lower and an 18% reduction in adjusted estimates. The blue area represents pre-implementation of CCP (SOC), green line indicates when CCP began implementation across study sites, and red indicates post-implementation of CCP. The trendline of NMR decrease is steeper than the World Bank NMR [15], showing the association of CCP towards reducing NMR. Median NMR from pre- and post-implementation of CCP calculated from states collectively is also presented, although this trendline is not steeper than the World Bank NMR. S5 Table outlines NMR rates as reported NFHS-4 and -5; S6 Table outlines dates for when pre- and post-data collection began.

## Discussion

In this large cohort, the family-centered Care Companion Program was associated with a reduced NMR compared to the pre-implementation period. In our previous studies, an increase in important neonatal behaviors has been associated with reduced mortality; improved adherence to key behaviors included uptake of dry cord care by 4%, skin-to-skin care practice by 78%, and 56% decrease in neonatal readmissions [13]. Here, the results suggest that participation in CCP may be associated with decreased risk of mortality. Our results show a viable strategy for improving neonatal outcomes following birth. Although the CCP program is focused on both maternal and neonatal behaviors postpartum, we did not see a reduction in maternal mortality. This study, however, was not designed to measure the relatively rare outcome of maternal mortality and notably the majority of the CCP content focuses on care of the neonate.

Due to a shortage of staff and a lack of parental knowledge about correct neonatal care practices, Family-Centered Care (FCC) is becoming a key opportunity to improve postnatal care [16]. Training physicians and nurses on delivering important medical information to patients as well as educating patients and caregivers are two key components of FCC [16]. FCC-based interventions have resulted in positive health outcomes in several conditions [13, 17–19]. Promotion of antenatal care and maternal health education is given to mothers and caregivers to improve key behaviors resulting in positive health outcomes [20–22]. Hospital-based FCC interventions focusing on awareness and improving the skills of parents and caregivers can be helpful in improving key behaviors and postnatal outcomes [13]. Similarly, FCC-based community interventions include sociocultural appropriate behavior modification techniques such as delivering health education and information to parents and caregivers, training birth attendants on identifying early danger signs, and training community health workers as master trainers to further train the family members on risky maternal and neonatal care practices were expected to reduce the neonatal mortality and morbidity burden on health systems [22–25].

The NMR per 1000 births observed in our study (pre: 41.3, post: 33.6) is much higher than the national average in the same time frame (24.9) [26]. This difference may be explained by the study population and CCP program at secondary and tertiary levels where Special Newborn Care Unit babies were also included. Focusing the family health education on a hospital setting with more high-risk babies likely had a greater impact on mortality.

Comparing the rate of change of NMR decrease from CCP compared to the larger general trends, the secular trend explains only a part of the difference we have seen. We did not see any new Maternal and Child Health initiatives occur in all intervention facilities in the 1.5 years between the pre- and post-data collection. However, programs like LaQshya [27] and non-health-related programs which took place in a few facilities could have contributed to the reduction. We believe that a large part of the difference seen is due to the direct training offered by CCP and the involvement of the whole family in behavior change.

The strengths of our study are the large sample size and representative data from multiple government facilities across 4 states. We adjusted for the cluster effect of the site using a logistic regression model to get a range around the point estimate. The pre-post study design allowed for a comparison group from the same hospitals, with similar populations seeking care, and similar settings reducing the variability.

There are five key limitations of our data. First, we could not measure the outcomes in the non-responders. We have compensated for this by calculating the NMR by including a denominator where we know the outcomes. Second, state-level estimates were not found to be significant, whereas the pooled estimates were. The tables for these analyses can be found in S4 Table. Third, for ethical reasons, we did not proceed with the survey when death was reported; thus, we have limited information to adjust for potential confounders, the cause, and timing of death post-discharge. Fourth, the demographics of the cohort for the completed surveys (without death) show statistically significant differences in the pre-and post-groups. There were typically older mothers, with more C-sections in the Post group, which may have a higher risk of mortality. We were unable to adjust for these differences in the Pre and Post groups, yet this group showed that mortality was reduced despite the Post group being at slightly higher risk. Finally, as an implementation research study design, we did not randomize individuals or clusters to receive the intervention and all data are self-reported.

## Conclusion

Postnatal training for caregiver skills could supplement health-systems’ existing efforts to reduce neonatal mortality. The estimated trend toward reduction in neonatal mortality over this short time span during CCP implementation is encouraging. Further studies should collect additional demographic details around deaths to understand at-risk populations. Further death reviews ascertaining the cause and timing of death as well as further exploring any causal pathways such as knowledge and practice of caregiving behaviors can help elucidate potential mechanisms involved in reducing mortality. Family-centered approaches to healthcare can help ensure proper patient care extends beyond hospitals into homes and lead to improved health outcomes. Noora Health plans to expand CCP’s footprint and evaluate the impact of caregiver education across condition areas and geographies.

## Supporting information

S1 Table Socio-demographic characteristics of respondents without a death. (DOCX)

S2 Table Neonatal mortality rates before and after introduction of the Companion Care Program (CCP) in four states in India. (DOCX)

S3 Table Unadjusted and hospital-level cluster adjusted estimates of maternal mortality and risk of mortality in post versus pre group. (DOCX)

S4 Table Neonatal mortality rates and risk ratios. (DOCX)

S5 Table Neonatal mortality rate across Karnataka, Punjab, Madhya Pradesh (MP), and Maharashtra reported from NFHS-4 and -5, against CCP associated rates. (DOCX)

S6 Table Pre and post data collection dates for each state. (DOCX)
