# Supplementary material for: Improving neonatal health with family-centered, early postnatal care: A quasi-experimental study in India
Source: PLOS Glob Public Health. 2023 May 25;3(5):e0001240. doi: 10.1371/journal.pgph.0001240 (PMC10212134; doi:10.1371/journal.pgph.0001240)
Supplement: S1 Table — (DOCX) [file pgph.0001240.s001.docx]

| **Age group** | **Pre**  **N(%)** | **Post**  **N (%)** | **P-value** |
| --- | --- | --- | --- |
| <=25 | 23240 (72.25) | 38489(66.35) | <0.001 |
| 26-35 | 8355 (26.0) | 18170 (31.3) |  |
| 36-58 | 173 (0.54) | 440 (0.76) |  |
| Don't know | 422 (1.3) | 921 (1.6) |  |
| **Education** | **Pre N (%)** | **Post N (%)** |  |
| No formal education | 3187 (9.9) | 6387 (11.01) | <0.0001 |
| 1-8 years of education | 10057 (31.24) | 15405 (26.55) |  |
| 9-11 years of education | 10163 (31.57) | 17656 (30.43) |  |
| Pre-University College Completed (12 years) | 4615 (14.34) | 10593 (18.26) |  |
| >=13 years | 4168 (12.95) | 7979 (13.75) |  |
| **Delivery type** | **Pre N (%)** | **Post N (%)** |  |
| Normal Vaginal Delivery | 21931 (68.13) | 35927 (61.92) | <0.0001 |
| C-Section Delivery | 10259 (31.87) | 22093 (38.08) |  |
| **Number of Babies** | **Pre N (%)** | **Post N (%)** |  |
| Single | 31926 (99.18) | 57542 (99.18) | <0.0001 |
| Twin/multiple | 264 (0.82) | 478 (0.82) |  |
| **Length of stay** | **Pre N (%)** | **Post N (%)** |  |
| <48 hours | 3523 (10.94) | 7266 (12.52) | < 0.001 |
| 48hr-7days | 23074 (71.68) | 40776 (70.28) |  |
| >7 days | 5577 (17.33) | 9928 (17.11) |  |
| Don't know | 16 (0.05) | 50 (0.09) |  |
| **First baby** | **Pre N (%)** | **Post N (%)** |  |
| No | 16251 (50.48) | 29058 (50.08) | 0.2 |
| Yes | 15939 (49.52) | 28962 (49.92) |  |
