# Supplementary material for: Improving neonatal health with family-centered, early postnatal care: A quasi-experimental study in India
Source: PLOS Glob Public Health. 2023 May 25;3(5):e0001240. doi: 10.1371/journal.pgph.0001240 (PMC10212134; doi:10.1371/journal.pgph.0001240)
Supplement: S2 Table — (DOCX) [file pgph.0001240.s002.docx]

| **Indicator** | **Pre-intervention (Standard of Care)**  **N=33599** | | **Post-intervention**  **(Care Companion Program)**  **N=60078** | | **Crude Risk Ratio (95% CI)** | **Cluster-adjusted Risk Ratio (95% CI)** |
| --- | --- | --- | --- | --- | --- | --- |
|  | Deaths (n) | Unadjusted Estimate (per 1000 live births) | Deaths (n) | Unadjusted Estimate (per 1000 live births) |  |  |
| NMR | 1386 | 41.26 | 2021 | 33.64 | 0.82 (0.76, 0.87) | 0.82 (0.71, 0.94) |
