# Supplementary material for: Improving neonatal health with family-centered, early postnatal care: A quasi-experimental study in India
Source: PLOS Glob Public Health. 2023 May 25;3(5):e0001240. doi: 10.1371/journal.pgph.0001240 (PMC10212134; doi:10.1371/journal.pgph.0001240)
Supplement: S3 Table — (DOCX) [file pgph.0001240.s003.docx]

| Indicator | Pre-intervention (Standard of Care) N=33599 | | Post-intervention  (Care Companion Program) N=60078 | | Crude Risk Ratio (95% CI ) | Cluster-adjusted Risk Ratio (95% CI ) |
| --- | --- | --- | --- | --- | --- | --- |
|  | Deaths (N) | Unadjusted Estimate (per 100,000 live births) | Deaths (N ) | Unadjusted Estimate (per 100,000 live births) |  |  |
| Maternal Mortality | 23 | 68.46 | 37 | 61.59 | 0.90  (0.53, 1.51) | 0.87  (0.53, 1.43) |
