# Supplementary material for: Improving neonatal health with family-centered, early postnatal care: A quasi-experimental study in India
Source: PLOS Glob Public Health. 2023 May 25;3(5):e0001240. doi: 10.1371/journal.pgph.0001240 (PMC10212134; doi:10.1371/journal.pgph.0001240)
Supplement: S4 Table — (DOCX) [file pgph.0001240.s004.docx]

**Appendix**

**S4 Table**: *Neonatal Mortality Rates and risk ratios, Model 1: adjusted for state and hospital-level clustering and Model 2: State specific mortality models, adjusted for hospital-level clustering*

| Indicator | Adj Pre^*^ N=33599 | | Adj CP ^1^ N=60078 | | Adj Risk Ratio | 95% CI |
| --- | --- | --- | --- | --- | --- | --- |
|  | Deaths (n) | NMR Estimate | Deaths (n) | NMR Estimate |  |  |
| Model 1: Model Adjusted for State and hospital level-clustering | | | | | | |
| Neonatal Mortality Rate  (per 1000 live births) | 1386 | 50.5 | 2021 | 41.3 | 0.93 | 0.82, 1.06 |
| Model 2: Model by state, adjusted for hospital-level clustering | | | | | | |
| Punjab: |  |  |  |  |  |  |
| Neonatal Mortality Rate  (per 1000 live births) | 70 | 29.6 | 606 | 31.2 | 1.06 | 0.84, 1.33 |
| Karnataka |  |  |  |  |  |  |
| Neonatal Mortality Rate  (per 1000 live births) | 341 | 26.1 | 468 | 22.9 | 0.88 | 0.69, 1.12 |
| Maharashtra: |  |  |  |  |  |  |
| Neonatal Mortality Rate  (per 1000 live births) | 30 | 45.8 | 192 | 33.2 | 0.73 | 0.49, 1.08 |
| Madhya Pradesh: |  |  |  |  |  |  |
| Neonatal Mortality Rate  (per 1000 live births) | 945 | 53.9 | 755 | 52.2 | 0.97 | 0.83, 1.13 |

The two models presented in the table are:

Model 1: Model with neonatal mortality as outcome and intervention group as primary independent variable and adjusted for state. The variability of estimates was adjusted for hospital-level clustering. This is reported above

Model 2: State-specific models with neonatal mortality as the outcome and intervention group as a primary independent variable. The variability of estimates was adjusted for hospital-level clustering within a state.
