# Supplementary material for: Improving neonatal health with family-centered, early postnatal care: A quasi-experimental study in India
Source: PLOS Glob Public Health. 2023 May 25;3(5):e0001240. doi: 10.1371/journal.pgph.0001240 (PMC10212134; doi:10.1371/journal.pgph.0001240)
Supplement: S5 Table — (DOCX) [file pgph.0001240.s005.docx]

**S5 Table**: *Neonatal Mortality rate across Karnataka, Punjab, Madhya Pradesh (MP), and Maharashtra reported from NFHS-4 and -5, against CCP associated rates*

| States | NFHS-4 (2015-2016) | NFHS-5 (2019-2021) | CCP associated NMR Pre-launch (2018-2020) | CCP associated NMR Post-launch (2018-2020) |
| --- | --- | --- | --- | --- |
| Karnataka | 18.5 | 15.8 | 26.1 | 22.9 |
| Punjab | 21.2 | 21.8 | 29.6 | 31.2 |
| Madhya Pradesh | 36.9 | 29 | 53.9 | 52.2 |
| Maharashtra | 16.2 | 16.5 | 45.8 | 33.2 |
