# Supplementary material for: Improving neonatal health with family-centered, early postnatal care: A quasi-experimental study in India
Source: PLOS Glob Public Health. 2023 May 25;3(5):e0001240. doi: 10.1371/journal.pgph.0001240 (PMC10212134; doi:10.1371/journal.pgph.0001240)
Supplement: S6 Table — (DOCX) [file pgph.0001240.s006.docx]

**S6 Table**: *Pre and post data collection dates for each state*

| State | Pre data Collection Start Date | Post data Collection Start Date |
| --- | --- | --- |
| Karnataka | 11th Oct 2018 | 26th Mar 2019 |
| Madhya Pradesh | 21st Nov 2018 | 24th July 2019 |
| Punjab | 10th Oct 2018 | 31st Oct 2018 |
| Maharashtra | 20th Mar 2019 | 29th Apr 2019 |
